# A modified CTAB method for the extraction of high-quality RNA from mono-and dicotyledonous plants rich in secondary metabolites

**DOI:** 10.1186/s13007-024-01198-z

**Published:** 2024-05-04

**Authors:** Tibor Kiss, Zoltán Karácsony, Adrienn Gomba-Tóth, Kriszta Lilla Szabadi, Zsolt Spitzmüller, Júlia Hegyi-Kaló, Thomas Cels, Margot Otto, Richárd Golen, Ádám István Hegyi, József Geml, Kálmán Zoltán Váczy

**Affiliations:** 1 Food and Wine Research Institute, Eszterházy Károly Catholic University, Eger, Hungary; 2 HUN-REN Centre for Ecological Research, Institute of Ecology and Botany, Vácrátót, Hungary; 3 Doctoral School of Biological Sciences, Hungarian University of Agriculture and Life Sciences, Gödöllő, Hungary; 4 Doctoral School of Environmental Sciences, Hungarian University of Agriculture and Life Sciences, Gödöllő, Hungary; 5 HUN-REN-EKKE Lendület Environmental Microbiome Research Group, Hungarian Research Network and Eszterházy Károly Catholic University, Eger, Hungary

**Keywords:** CTAB, RNA extraction, qRT-PCR, Polysaccharide and polyphenolic rich plants

## Abstract

**Background:**

High-quality RNA extraction from woody plants is difficult because of the presence of polysaccharides and polyphenolics that bind or co-precipitate with the RNA. The CTAB (cetyl trimethylammonium bromide) based method is widely used for the isolation of nucleic acids from polysaccharide-rich plants. Despite the widespread use of the CTAB method, it is necessary to adapt it to particular plant species, tissues and organs. Here we described a simple and generalized method for RNA isolation from mature leaf tissues of several economically important woody (17) and herbaceous plants (2) rich in secondary metabolites. High yields were achieved from small amount (up to 50 mg) of plant material. Two main modifications were applied to the basic protocol: an increase in β-mercaptoethanol concentration (to 10%v/v) and the use of an effective DNase treatment. As opposed to similar studies, we tried to describe a more detailed protocol for isolating RNA, including the exact quantity and concentration of the reagents were used.

**Results:**

Our modified CTAB method is proved to be efficient in extracting the total RNA from a broad range of woody and herbaceous species. The RNA yield was ranged from 2.37 to 91.33 µg/µl. The A_260_:A_280_ and A_260_:A_230_ absorbance ratios were measured from 1.77 to 2.13 and from 1.81 to 2.22. The RIN value (RNA Integrity Number) of the samples fell between 7.1 and 8.1, which indicated that a small degree of RNA degradation occurred during extraction. The presence of a single peak in the melt curve analyses and low standard errors of the Ct values of replicated measurements indicated the specificity of the primers to bind to the cDNA.

**Conclusions:**

Our RNA isolation method, with fine-tuned and detailed instructions, can produce high quality RNA from a small amount of starting plant material that is suitable for use in downstream transcriptional analyses. The use of an increased concentration of the reducing agent β-mercaptoethanol in the extraction buffer, as well as the application of DNaseI-treatment resulted in a method suitable for a wide range of plants without the need of further optimalization, especially in *Rhus typhina* (Staghorn sumac), for which molecular-genetic studies have not yet been sufficiently explored.

**Supplementary Information:**

The online version contains supplementary material available at 10.1186/s13007-024-01198-z.

## Introduction

An important prerequisite of molecular genetic analyses is the availability of high-quality RNA [1–4]. In plants, isolation of this molecule is often difficult, as tissues of different organs may contain significant amounts of secondary metabolites (polysaccharides and polyphenols) [5]. These compounds can only be removed with great difficulty during extraction [6, 7]. It has been described that phenolic compounds readily oxidize quinones, which, when bound to RNA, render it useless for basic processes, such as reverse transcription and cDNA library construction [8, 9]. These compounds can be found in almost all the tissues of woody dicotyledonous plants [10, 11]. The most common commercial RNA isolation kits use an acid guanidinium thiocyanate-phenol-chloroform extraction reagent, which could lead to RNA of poor quality [12]. Therefore, variants of the CTAB (cetyl trimethylammonium bromide) based method are widely used for nucleic acid isolation from polysaccharide-rich plants to overcome the above limitations [2, 12–16]. A typical CTAB extraction buffer contains CTAB, polyvinylpyrrolidone (PVP), sodium chloride and β-mercaptoethanol, each of which plays an important role in nucleic acid extraction from polysaccharide-rich samples [2]. Despite the widespread use of the CTAB method, it is necessary to adapt this method to a particular plant species and the fine-tuning of the isolation steps is also inevitable.

Here we report an easily applicable method for RNA isolation from mature leaf tissues of seventeen economically important woody (including grapes: *Vitis vinifera* L.) and two economically important herbaceous plants (banana: *Musa* sp. and bread wheat: *Triticum aestivum* L.) with a high secondary metabolites content. The advantages of isolation from leaves include fast and easy sample collection, the presence of large amounts of RNA in the organ and the possibility to study not only molecular-genetic changes in physiological processes but also plant-microbe interactions. While fine-tuning the method, we aimed to extract RNA from mature leaves, as such leaves contain an increased amount of polyphenols, tannins and polysaccharides [17], therefore, RNA extraction is possible also when younger, developing leaves and shoots are not available. In contrast to most methods, we aimed (1) to achieve the highest possible yield of the total RNA from a small sample (up to 50 mg), (2) to increase the concentration of β-mercaptoethanol (to 10%v/v) (3) to use an effective DNase treatment and (4) to describe a more detailed protocol for isolating RNA, including the exact quantity and concentration of the reagents were used. RNA yield, purity (absorbance ratios A_260_:A280 and A_260_:A_230_) and integrity (RIN – RNA Integrity Number) were determined for each sample. In addition, isolated RNA was assessed for use in qRT-PCR assays.

## Materials and methods

### Plant materials

To verify the efficiency of the method, 17 woody and 2 herbaceous plant species were tested (Table 1).

**Table 1:** The list of the plant materials was tested

Common name	Scientific name
Apple	*Malus domestica* Borkh.
Black elderberry	*Sambucus nigra* L.
European ash	*Fraxinus excelsior* L.
Wild cherry	*Prunus avium* L.
White birch	*Betula pendula* Roth.
European pear	*Pyrus communis* L.
Apricot	*Prunus armeniaca* L.
Staghorn sumac	*Rhus typhina* L.
Blackthorn	*Prunus spinosa* L.
Common lilac	*Syringa vulgaris* L.
Quince tree	*Cydonia oblonga* M.
Dog rose	*Rosa canina* L.
Black locust tree	*Robinia pseudoacacia* L.
Arizona walnut	*Juglans major* Torr.
Grape	*Vitis vinifera* L.
Banana	*Musa* sp.
Passion fruit	*Passiflora eduli*s Sims.
Lemon	*Citrus* sp.
Bread wheat	*Triticum aestivum* L.

In the case of leaf samples of *Musa* sp., *P*. *edulis* and *Citrus* sp. were collected from the greenhouse of Food and Wine Research Institute, Eszterházy Károly Catholic University, Eger. The leaf tissues of other species were collected from native plants around Eger. Leaf samples were kept at -80 °C until use.

Grape is one of the most important economic woody crops all over the world. All tissues of this plant are also rich in secondary metabolites. Furthermore, wheat is one of the most significant monocot plants in human food supply. For these reasons, a more detailed RNA quality control study (qRT-PCR analysis) was only performed with these species.

### Solutions and reagents

- Composition of 2% CTAB extraction buffer and recipe for 100 ml:

2%m/v CTAB (Cetyltrimethylammonium bromide, CAS No: 57-09-0, Carl Roth GmbH, Germany) – 2 g.

2 M NaCl (CAS No: 7647-14-5, Fluka Analytical, USA) – 11.69 g.

100 mM Tris-HCl (CAS No: 1185-53-1, Biochem Chemopharma, France) – 1.575 g.

20 mM EDTA (Ethylenediaminetetraacetic acid, CAS No: 6381-92-6, Reanal, Hungary) – 0.75 g.

2.5%m/v PVP-40 (Polyvinylpyrrolidone, CAS No: 9003-39-8, Sigma-Aldrich, USA) – 2.5 g.

pH = 8 (adjusted by 1 M NaOH).

10%v/v β-mercaptoethanol (CAS No: 60-24-2, ThermoScientific, USA) – 10 ml.

ddH_2_O (double distilled water).

- Chloroform : isoamyl alcohol (24:1 v/v) (50 ml):

48 ml chloroform.

2 ml isoamyl alcohol.

- 8 M LiCl (50 ml):

16.96 g of powdered LiCl (CAS No: 7447-41-8, Fluka Analytical, USA) dissolved in ddH_2_O (double distilled water).

- 80% Ethanol.
- Isopropanol.
- QIAGEN RNase-Free DNase Set (Hilden, Germany) to remove possible DNA contamination.

The components in the extraction buffer, excluding β-mercaptoethanol, were mixed and heated for 30 min at 100 °C for sterilisation.

### Protocol for isolation of RNA

900 µL of extraction buffer (10%v/v of β-mercaptoethanol added just before use) were pre-heated at 65 °C in a microcentrifuge tube for 10 min. 30–40 mg (no more than 50 mg) of sample (leaf tissue) were grounded to a fine powder in liquid nitrogen using a mortar and a pestle. The pre-heated extraction buffer was added to powder with subsequent grinding to make a homogenous mixture. Then, the mixture was transferred to a sterile 2 ml microcentrifuge tube and was incubated at 65 °C for 10 min, with the rotation of the tubes every two minutes. An equal (900 µL) volume of chloroform : isoamyl alcohol (24:1 v/v) was added and the tube was inverted vigorously and centrifuged at 15.000 rpm for 10 min at 4 °C. A 500 µL volume of the upper aqueous phase was transferred to a new sterile microcentrifuge tube and equal volume of chloroform : isoamyl alcohol (24:1 v/v) was added and centrifuged again at 15.000 rpm for 10 min at 4 °C. 300 µL of the upper phase was transferred to a new sterile microcentrifuge tube and 8 M LiCl was added in an equal volume. The mixture was rotated several times, spinned and was incubated at -20 °C for 24 h. Then, RNA was selectively pelleted by a centrifugation at 15.000 rpm for 45 min at 4 °C. The pellet was washed with 500 µL ice-cold ethanol (80%v/v) and centrifuged at 15.000 rpm for 5 min at 4 °C. The supernatant was carefully removed using a pipette and the tube was centrifuged again (collecting any remained alcohol in the bottom of the tube). The rest of the alcohol was removed with a pipette and the precipitate was dried under a laminar box for 3–5 min.

*DNase treatment*: after the drying of the nucleic acid pellet, 175 µL of RNase-free water was added to the tube and incubated at 50 °C for 2 min. Then, 20 µL of RNase-free buffer (RDD) and 5 µL of RNase-free DNase I enzyme were added, vortexed briefly (∼ 1 s) and incubated at 37 °C for 20 min, followed by another incubation at 60 °C for 10 min (DNase inactivation). Then 200 µL of isopropanol was added and mixed vigorously. The tube was kept at -20 °C for 60 min, followed by a centrifugation at 15.000 rpm for 50 min (at 4 °C). The pellet was washed with 500 µL ice-cold ethanol (80%v/v) and centrifuged at 15.000 rpm for 5 min at 4 °C. The supernatant was removed carefully (using a pipette) and the centrifugation was repeated to collect any remaining alcohol in the bottom of the tube. The rest of the alcohol was removed with a pipette and the pellet was dried under a laminar box for 3–5 min. The pellet was resuspended in 30 µL of RNase-free water.

### RNA assessment and qRT-PCR analysis

Purity and concentration of RNA was measured by determining the absorbance of the sample between 260 and 230, as well as 260 and 280 nm wavelengths using an UV-Vis spectrophotometer (NanoDrop™ 2000, Thermo Scientific, Massachusetts, USA). The integrity analysis of RNA was carried out by Agilent RNA 6000 Nano LabChip® (Agilent 2100 Bioanalyzer, Califronia, USA). The cDNA synthesis was performed using 1.0 µg of the total RNA with the RevertAid First Strand cDNA Synthesis Kit (Thermo Fisher Scientific Inc, Waltham, USA) applying the standard protocol provided by the company. Determination of cycle threshold (Ct) values for each housekeeping gene was done using the generic gene specific primers listed in Table S1.

Quantitative real-time PCR measurements were carried out using three biological and two technical replicates in a Corbett RotorGene 6000 device (Qiagen Ltd, Hilden, Germany), applying the sybr‐green technology of the company (QuantiNova RT-PCR Kit, Qiagen Ltd, Hilden, Germany). PCR amplification conditions were 95 °C for 6 min, 40 cycles of 95 °C for 20 s, 62 °C for 30 s and 72 °C for 50 s. The final extension was 72 °C for 10 min.

### Statistics

Data represent the mean ± SE (Standard Error). The boxplots were carried out by SPSS 23.0 (IBM Data Science Community) program package.

## Results and discussion

Our version of the so-called CTAB method proved to be efficient in extracting both woody and herbaceous total RNA, indicated by the A_260_:A_280_ and A_260_:A_230_ ratios measured from 1.77 to 2.13 and from 1.81 to 2.22 (Table 2, Fig. S1).

**Table 2:** Nano-spectrophotometric analysis of RNA yield, purity (A_260_:A_280_ and A_260_:A_230_) and Integrity Numbers (RIN) using NanoDrop™ 2000 and Bioanalyzer (Agilent 2100) for the total RNA isolated using the modified CTAB method from mature leaf tissue of woody and herbaceous plants. ^a^ Values represent the mean ± SD from at least three technical replicates

Species	RNA yield (µg/50 mg FW)	A_260_:A_280_ ratio	A_260_:A_230_ ratio	RIN
*Malus domestica*	48.22±2.73	2.11±0.02	2.20±0.07	7.20
*Fraxinus excelsior*	64.43±7.33	2.13±0.01	2.22±0.01	7.30
*Sambucus nigra*	38.48±4.72	2.08±0.03	2.19±0.01	7.60
*Prunus avium*	47.105±8.69	2.09±0.03	2.15±0.02	7.50
*Betula pendula*	91.33±3.41	2.09±0.04	2.04±0.01	7.80
*Prunus armeniaca*	12±0.75	2.07±0.06	2.11±0.03	7.60
*Pyrus communis*	52.40±10.36	2.07±0.05	2.11±0.04	7.70
*Rhus typhina*	15.47±7.98	2.07±0.01	2.09±0.02	7.60
*Prunus spinosa*	56.94±9.42	2.09±0.04	2.05±0.03	7.40
*Cydonia oblonga*	26.93±3.48	2.06±0.02	2.08±0.01	7.60
*Syringa vulgaris*	8.62±6.03	2.05±0.04	2.06±0.02	7.90
*Rosa canina*	44.89±5.14	1.77±0.03	1.81±0.02	7.10
*Robinia pseudoacacia*	2.37±1.03	2.05±0.07	1.92±0.14	7.60
*Vitis vinifera*	32.13±10.44	2.05±0.01	2.08±0.03	8.10
*Juglans major*	14.35±0.3	2.07±0.03	2.09±0.01	7.80
*Triticum aestivum*	33.86±9.61	1.99±0.05	2.02±0.03	7.40
*Musa sp.*	3.40±1.9	2.07±0.09	2.22±0.05	7.10
*Passiflora edulis*	7.48±2.37	2.05±0.07	2.14±0.09	7.80
*Citrus sp.*	4.06±1.69	2.03±0.05	2.12±0.11	8.10

RNA absorbs UV light maximally at 260 nm, whereas proteins absorb it at 280 nm and other contaminants including carbohydrates, phenol, and aromatic compounds generally absorb it around 230 nm. Therefore, the A_260_:A_280_ and the A_260_:A_230_ ratios are often used to assess RNA sample purity. Generally, samples with ratio values in the range of ∼ 1.8–2.0 indicate high purity RNA [2]. The integrity analysis of the RNA was carried out using Agilent 2100 BioAnalyzer, which provides the 28 S/18S ratio and the RIN for RNA quality control. The 28 S/18S ratio was between 1.3 and 4.0, which is close to the ideal value of 2.0 or higher [2]. The RIN value (RNA Integrity Number) of the samples was between 7.1 and 8.1, which indicated that a small degree of RNA degradation occurred during extraction (Table 2, Fig. S2).

**Fig. 1:**
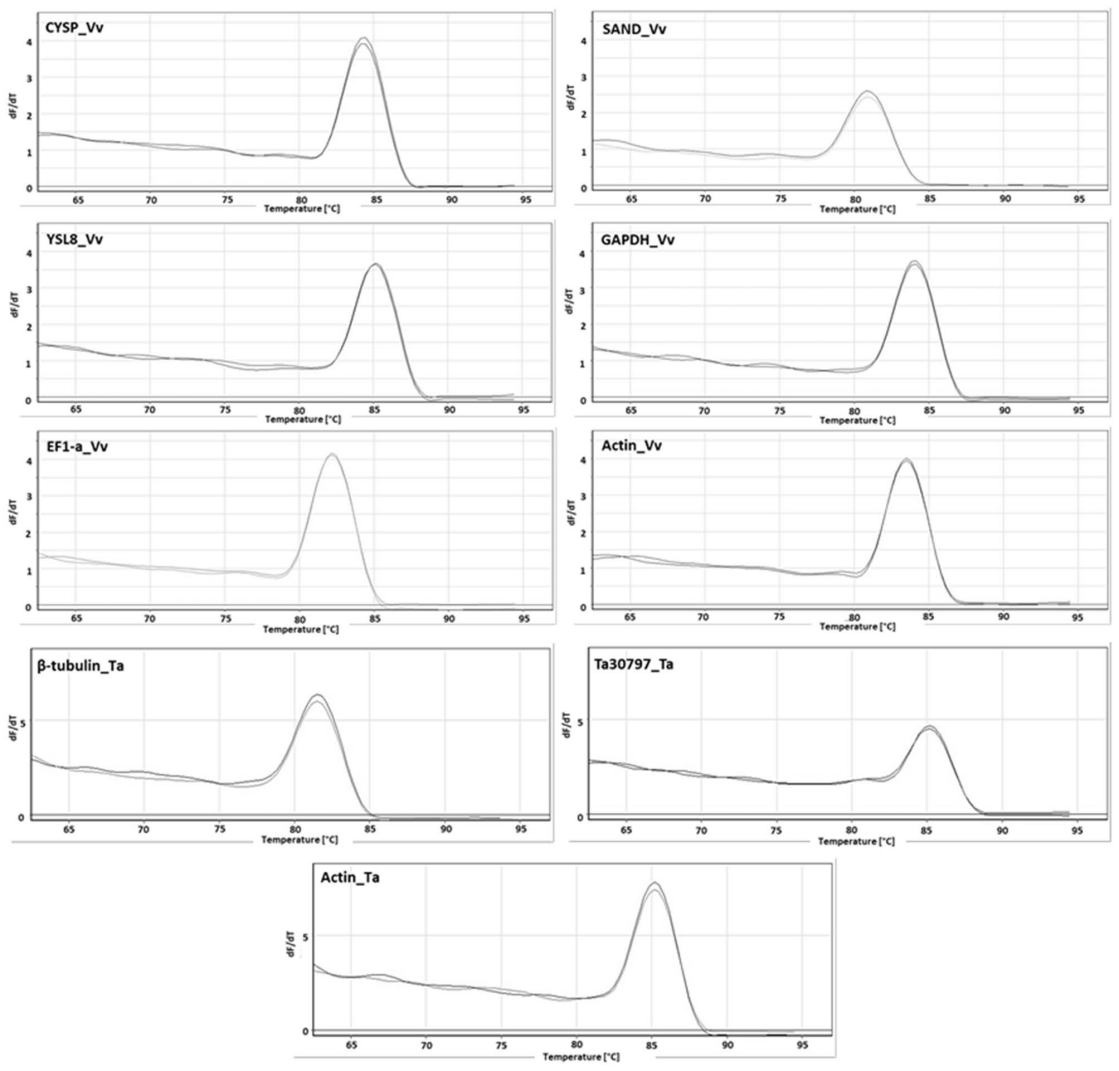
qRT-PCR melt curves of the nine housekeeping genes. dF/dT represents change in fluorescence level (positive or negative) with respect to per unit change in temperature Vv: *Vitis vinifera*; Ta: *Triticum aestivum*

**Fig. 2:**
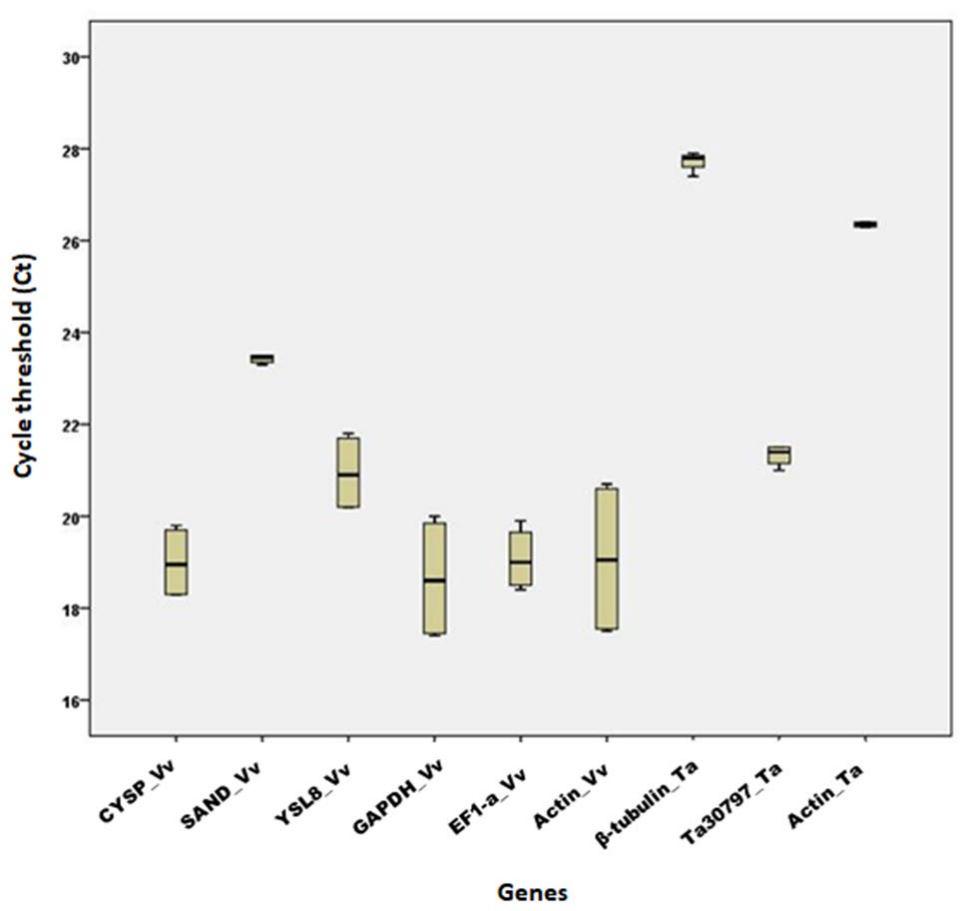
Graph of cycle thresholds (Ct) of selected housekeeping genes using cDNA from RNA isolated from *Vitis vinifera* L. (Vv) and *Triticum aestivum* L. (Ta) leaves and amplified using qRT-PCR. Error bars indicate standard errors

The RNA yield was ranged from 2.37 to 91.33 µg/µl (from 50 mg leaf tissue) (Table 2). The suitability of the isolated RNA to downstream processes was determined by transcriptional analyses of nine housekeeping genes (*CYSP*, *YSL8*, *Actin*, *SAND*, *EF1*-*α*, *GAPDH* for *V*. *vinifera* and *β-tubulin*, *Ta30797*, *Actin* for *T*. *aestivum*) using quantitative real-time PCR (qRT-PCR) with three biological and two technical replicates (Table S1). The presence of a single peak in the melt curve analyses (Fig. 1), as well as the low standard errors between the measured Ct values (Fig. 2) indicated the specificity of the primers to bind to the cDNA.

**Fig. 3:**
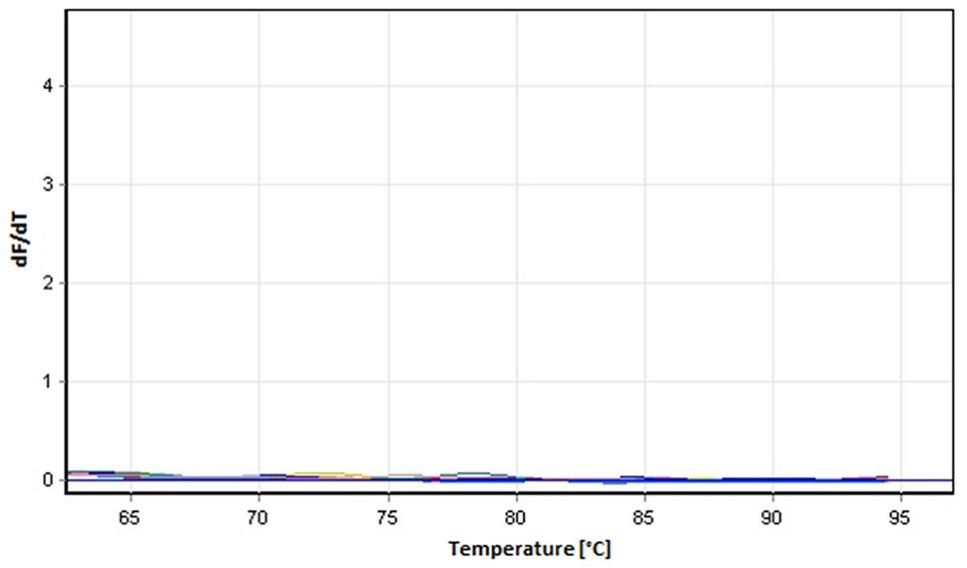
qRT-PCR melt curves of the nine housekeeping genes with RNA samples (not applied reverse transcribed) using modified CTAB isolation method demonstrating that RNA samples contain DNA below detection levels. dF/dT represents change in fluorescence level (positive or negative) with respect to per unit change in temperature

No interference of PCR inhibitors or other contaminants, such as gDNA, was observed (Fig. 1). This latter finding was confirmed by performing qRT-PCR analyses of nine housekeeping genes on the RNA that was not reverse transcribed (NO-qRT-PCR). No melt curves were observed when NO-qRT-PCR controls were used as templates, indicating that our method was able to isolate the total RNA that is free of gDNA contamination, demonstrating the effectiveness of DNase treatment (Fig. 3).

The main modifications of the protocol described here, compared to the original method [18], were (1) a reduction of sample amount (up to 50 mg fresh weight), (2) an increase in β-mercaptoethanol concentration (10%v/v of total buffer volume) and (3) using an effective Dnase treatment with QIAGEN Rnase-Free Dnase Set (Hilden, Germany) to remove possible DNA contamination. Most methods use significantly higher amounts of plant material for the total RNA extraction [1, 11–15, 19, 20]. With our method, we have demonstrated that a relatively small amount of sample (0.05 g) can be used to extract high-quality (RIN: 7.1–8.1) and sufficient amounts of RNA (in case of *B*. *pendula* ∼ 91 µg). β-Mercaptoethanol is a strong reducing agent and is able to break disulfide bonds. According to most protocols, using 1–2%v/v β-mercaptoethanol was effective, but Ouyang et al. [21] demonstrated degraded RNA from different tissues of *Neolamarckia cadamba*, even at 5%v/v β-mercaptoethanol. Therefore, an increase of β-mercaptoethanol concentration in the RNA extraction method can reduce polyphenol oxidation and inactivate ribonucleases [18]. Since the taxonomically diverse plants tested contain different levels of secondary metabolites, our experience showed that it was necessary to increase the concentration of β-mercaptoethanol significantly. The same method could be used for DNA isolation (isopropanol precipitation instead of LiCl followed by the removal of RNA contamination by DNase-free RNase A).

## Conclusions

Our RNA isolation method, with fine-tuned and detailed instructions, can produce high quality RNA from a small amount of starting plant material that is suitable for use in downstream transcriptional analyses. This method has been tested on several species of woody and on a few herbaceous plants, which are rich in secondary metabolites, and has been validated by qRT-PCR. The use of an increased concentration of the reducing agent β-mercaptoethanol in the extraction buffer, as well as the application of DNaseI-treatment, resulted in a method suitable for a wide range of plants without the need of further optimization, especially in *Rhus typhina* (Staghorn sumac), for which molecular-genetic studies have not been sufficiently explored yet.

### Electronic supplementary material

Below is the link to the electronic supplementary material.

Supplementary Material 1

Supplementary Material 2

Supplementary Material 3

## Data Availability

The results of this manuscript do not contain any newly generated sequence data or gene expression data that could be deposited in an international archive. Data is provided within the manuscript or supplementary information files. The results and technical details present in our methodology manuscript are available for researchers via the corresponding author.
