# Supplementary figures and images for: A modified CTAB method for the extraction of high-quality RNA from mono-and dicotyledonous plants rich in secondary metabolites
Source: Plant Methods. 2024 May 4;20:62. doi: 10.1186/s13007-024-01198-z (PMC11069240; doi:10.1186/s13007-024-01198-z)

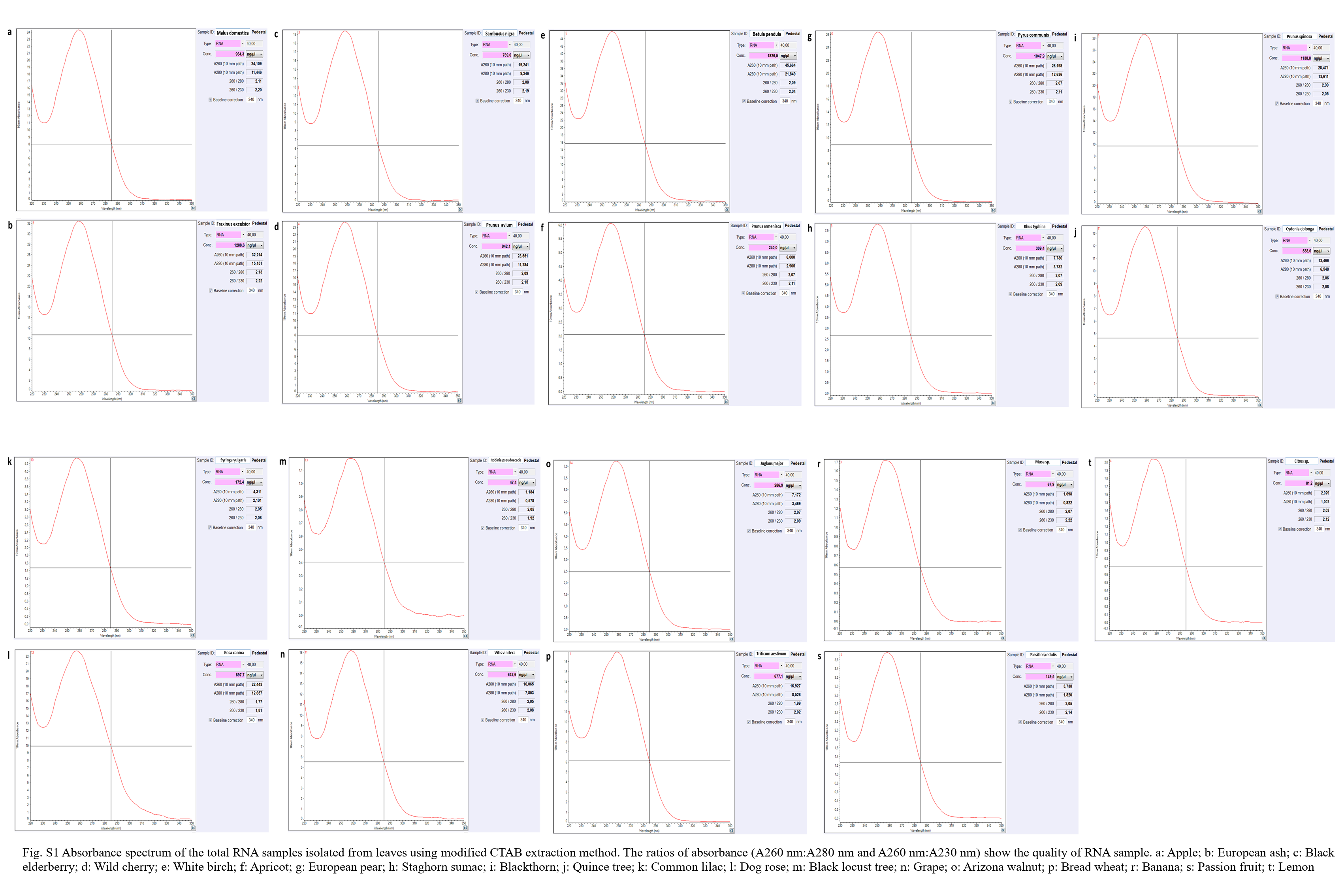

Supplement: Supplementary file 1 — Supplementary Material 1 [file 13007_2024_1198_MOESM1_ESM.tif]

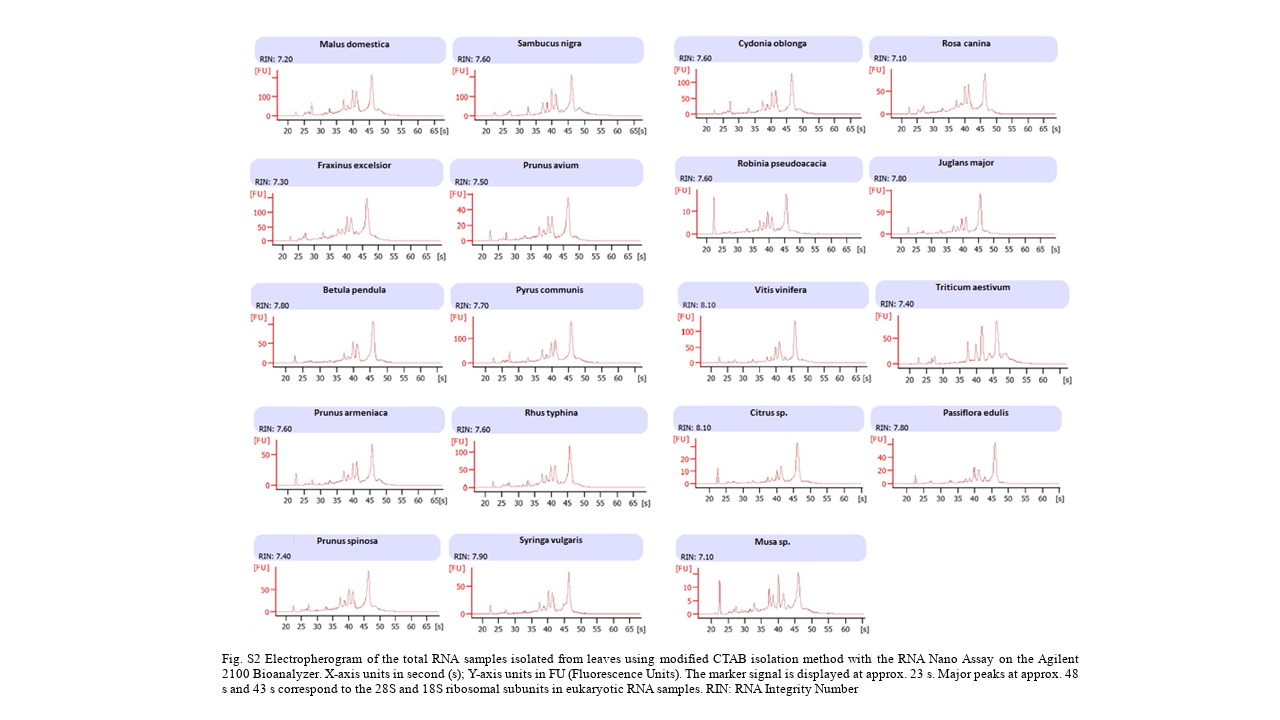

Supplement: Supplementary file 2 — Supplementary Material 2 [file 13007_2024_1198_MOESM2_ESM.tif]

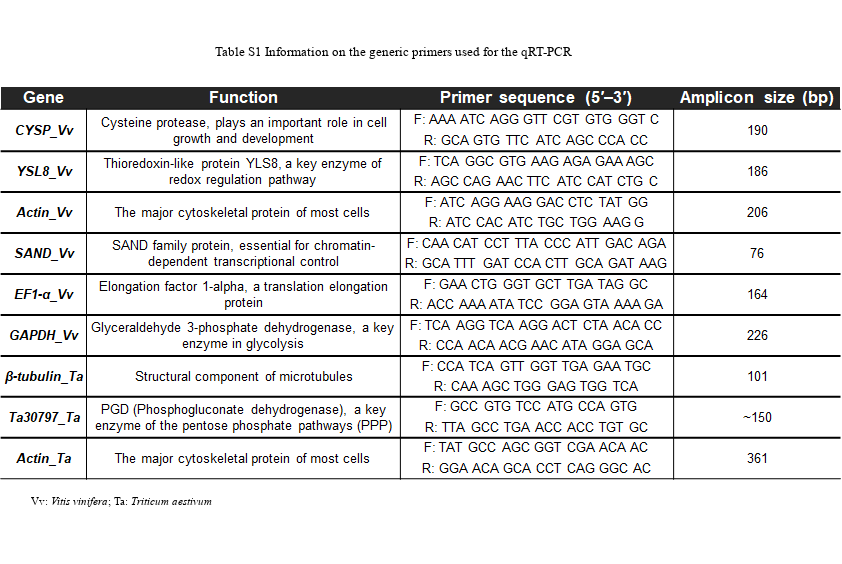

Supplement: Supplementary file 3 — Supplementary Material 3 [file 13007_2024_1198_MOESM3_ESM.tif]
